# Efficacy of Vaccinations

**Published:** 1883-07

**Authors:** 


EFFICACY OF VACCINATION.—During the epidemic of small pox in Baltimore City last winter, the entire class of the Dental Department of the University of Maryland, with *one* exception, were vaccinated by the different members of the Faculty.

The student who refused to be vaccinated was the only case of the disease among a class numbering sixty-seven members, and although he recovered deeply marked by the confluent form of the affection, his general health has greatly improved, and his weight has considerably increased. No better evidence of the efficacy of vaccination as a preventive of the disease, is needed.

